# Temporal Trends of Melanoma and Non-Melanoma Skin Cancer Mortality Rates in Hungary Between 1980 and 2020

**DOI:** 10.3390/cancers18162533

**Published:** 2026-08-07

**Authors:** Ágnes Stier, Anna Páldy

**Affiliations:** 1 Health Sciences Division, Doctoral College, Semmelweis University, 1085 Budapest, Hungary; stier.agnes@phd.semmelweis.hu; 2 Department of Environmental Health Laboratory, National Center for Public Health and Pharmacy, 1097 Budapest, Hungary

**Keywords:** melanoma, skin cancer, mortality, Hungary, epidemiology, trends

## Abstract

**Background**: Long-term mortality trends for melanoma and non-melanoma skin cancer (NMSC) in Hungary have not been comprehensively assessed. **Methods**: We conducted a retrospective nationwide study of deaths registered in Hungary between 1980 and 2020 among individuals aged 35 years or older, where melanoma (ICD-10 C43) or NMSC (ICD-10 C44) was recorded as the primary cause of death. Annual crude death rates (CDRs; overall and age-specific 35–64 and ≥65 years) and age-standardised death rates (ASDRs) per 100,000 population were calculated by sex. Trends were analysed using Joinpoint regression. **Results**: Overall, 18,857 skin cancer deaths, including 12,026 melanoma and 6831 NMSC deaths, were identified. Melanoma mortality was consistently higher in men. Among men, melanoma ASDR increased until 1994 and then stabilised, while crude mortality among older men continued to increase. Among women, the ASDR of melanoma increased until 1994 and subsequently declined. Crude mortality decreased after 2005 in women aged 35–64 years. Although NMSC mortality was lower than melanoma mortality, NMSC accounted for 36.2% of skin cancer deaths. In both sexes, NMSC mortality was concentrated in adults ≥ 65 years. In older women, crude NMSC mortality increased again after the mid-2000s, whereas ASDR remained broadly stable. **Conclusions**: Melanoma mortality trends in Hungary partially stabilised or improved after the mid-1990s, particularly in age-standardised analyses, but mortality remained high among older adults, especially among men. NMSC represented a substantial proportion of skin cancer deaths, with the burden concentrated in adults ≥ 65 years, supporting the inclusion of NMSC in routine mortality surveillance and disease burden assessments.

## Simple Summary

This study reports the first long-term national analysis of melanoma and non-melanoma skin cancer (NMSC) mortality trends in Hungary between 1980 and 2020. It provides a detailed comparison of mortality trends by sex and age group, with different epidemiological trends for the two types of malignancies. Melanoma mortality demonstrated a positive downwards trend starting in the mid-1990s, presumably indicating improvements in early detection and treatment, whereas NMSC mortality remained a significant burden, particularly among the elderly. These results underscore the different public health challenges posed by melanoma and NMSC and the need to include NMSC in routine mortality surveillance and disease burden estimation to inform prevention and healthcare planning.

## 1. Introduction

Skin cancer poses significant pressure on healthcare. It can be divided primarily into non-melanoma skin cancer (NMSC) and melanoma. NMSC, including basal cell carcinoma (BCC), squamous cell carcinoma (SCC), among others, is the most diagnosed cancer worldwide. However, many cancer registries often exclude NMSC or fail to report BCC and SCC separately [1,2]. Urban et al. (2021) [3] reported, that incident cases globally increased by 77.4% and 309.7% for BCC and SCC, respectively, between 1990 and 2017 [3]. In 2022, NMSC ranked third after lung and prostate/breast cancer in terms of the total number of new cases diagnosed (744,792 cases in men and 489,803 cases in women). It can be assumed that skin cancer mortality is often not accurately recorded since statistical analyses exclude NMSC [4,5]. Nevertheless, despite NMSC being less fatal, owing to its high incidence, more deaths are attributed to NMSC than to melanoma globally [6].

Melanoma ranked 21st globally in terms of cancer incidence and third in terms of skin cancer incidence between 1990 and 2017 (160.4% increase) [3]. In 2022, 331,647 new melanoma cases were reported globally, making it the 14th most common cancer, causing a total of 206,839 deaths [7].

Urban et al. (2021) [3] reported a higher disability-adjusted life years (DALY) in the less-researched central and eastern European regions for melanoma and NMSC. Melanoma DALY rates were more than twice the global average DALY rate in these regions [3]. This finding is not surprising since overall cancer mortality is 10% higher than the EU average in this region [8].

Melanoma was among the top ten most frequently diagnosed cancers in Hungary, which belongs to this region, in the past decade, and prompted research interest. Parrag et al. (2022) reported an improvement in the melanoma survival rate from 2001 to 2019 [9]. This was also confirmed for the period 2011–2018 by Várnai et al. (2022) and for the period 2001–2018 by Gubán et al. (2025) [10,11]. Koczkodaj et al. (2023) [12] reported a decline in mortality rates from 1960 to 2020, when public databases for the EU-27 were used. The age-standardised melanoma mortality rate in Hungary was lower for men (11.2%) than for women (30.0%) among individuals aged 75 years or older [12]. NMSC incidence increased in both sexes from 2001 to 2015 [13]. Mortality trends in NMSC in Hungary have not been studied recently.

We aimed to provide a nationwide assessment of skin cancer mortality in Hungary from 1980 to 2020, on the basis of death certificates listing these cancers as the primary cause of death (COD). To our knowledge, this is the first long-term study to examine trends in the mortality of skin cancer patients in Hungary.

## 2. Methods

This retrospective study analysed melanoma and NMSC mortality in Hungary from 1980 to 2020. Furthermore, to mitigate the impact of the COVID-19 pandemic, we decided to end the study period with the year 2020. We examined the temporal trends of the crude death rates (CDRs) and age-standardised death rates (ASDRs) of patients with melanoma and NMSC. The Hungarian Central Statistical Office (HCSO) provided mortality data through its on-site research room. Access to the research room is subject to prior authorisation (contract number: KSH/ADKI/658-5/2023).

Eligible cases were defined as those for which the primary COD was melanoma (ICD-10 C43) or NMSC (ICD-10 C44). The HCSO provided anonymised, aggregated data by sex, age (birth year), citizenship, or native language. The latter variables were used to exclude non-eligible non-Hungarian citizens (*N* = 188). Age was grouped into bands from 35–44 to 85+. The number of individuals under 35 years of age was negligible.

The HCSO provided the mortality data after harmonising the original records to the ICD-10 coding system [14]. HCSO supplied the results of our analysis after data protection, in accordance with the General Data Protection Regulation (GDPR) (Regulation (EU) 2016/679). Patient identification was not possible. The data-protected tables were suppressed if a cell contained fewer than two cases (values 1 or 2). HCSO released true zeros. Melanoma cases had only 2 suppressed cells in the 35–44-year-old age group over the 40-year period, and all other age groups were complete. Similarly, NMSC needed suppression primarily in the 35–44-year-old group (*N*(men) = 24 and *N*(women) = 34). Since this suppression was not random, the authors decided to use simple substitution with a midpoint value of 1. To control for bias, sensitivity analysis was performed on the ASDR calculations of the NMSC, using the alternative value of 2, as an upper bound. Since zeros represented true zeros, we concluded that a sensitivity analysis with a value of zero was not needed.

Because NMSC frequently occurs as a comorbidity in older patients and may not be selected as the underlying COD, we assessed the validity of COD coding as follows: mortality counts based on the underlying (primary) COD were compared with those based on multiple COD records. Multiple COD data included all death certificates on which melanoma or NMSC was recorded, irrespective of whether it was assigned as the underlying COD. Absolute differences between primary and multiple COD counts were calculated by age group and sex.

We calculated CDRs per 100,000 population for the entire country between 1980 and 2020, stratified by sex and using midyear population data from HCSO. We calculated the annual CDR for individuals ≥ 35 years (hereafter referred to as the overall CDR) and for the 35–64-year-old and 65 years or older populations to determine whether the trends were consistent across these two age groups. We calculated the annual ASDR using direct standardisation and 10-year age bands for a population of 100,000 population using the European Standard Population (ESP 1976) [15]. All rates mentioned later in the text are expressed per 100,000.

We performed a Joinpoint time trend analysis of the natural logarithms of CDR and ASDR (version 5.0.2, National Cancer Institute at the National Institutes of Health, USA, available at: https://surveillance.cancer.gov/joinpoint/, accessed on 13 April 2026). We excluded women aged 35–64 with NMSC diagnosis from the model because the case numbers were negligible in this age group (minimum 2, maximum 22). The Joinpoint trend analysis estimates the annual percent change (APC) with 95% confidence intervals (CIs) by a best-fit logarithmic linear model [16]. Joinpoint uses a modified/weighted form of the Bayesian Information Criterion (BIC) during model selection, and an uncorrelated-error model was chosen. The model allowed a maximum number of joinpoints (maximum 7). The constant-variance (homoscedasticity) assumption was selected for the analysis, because no substantial differences in variance across the study period were observed, although some year-to-year variability remained. We interpreted only significant APCs in our analysis. The default CI method in the Joinpoint software (version 5.0.2) is the empirical quantile CI method, which may be less stable in years with small case numbers due to the increased variability.

We obtained ethical approval from the Medical Research Council, case number BM/21054-1/2023. The study was performed from June 2023 to February 2024.

## 3. Results

There were 18,857 skin cancer cases in Hungary between 1980 and 2020 among the population aged 35 years or older. This included 6525 men and 5501 women with melanoma, and 3451 men and 3380 women who died of NMSC.

**Figure 1:**
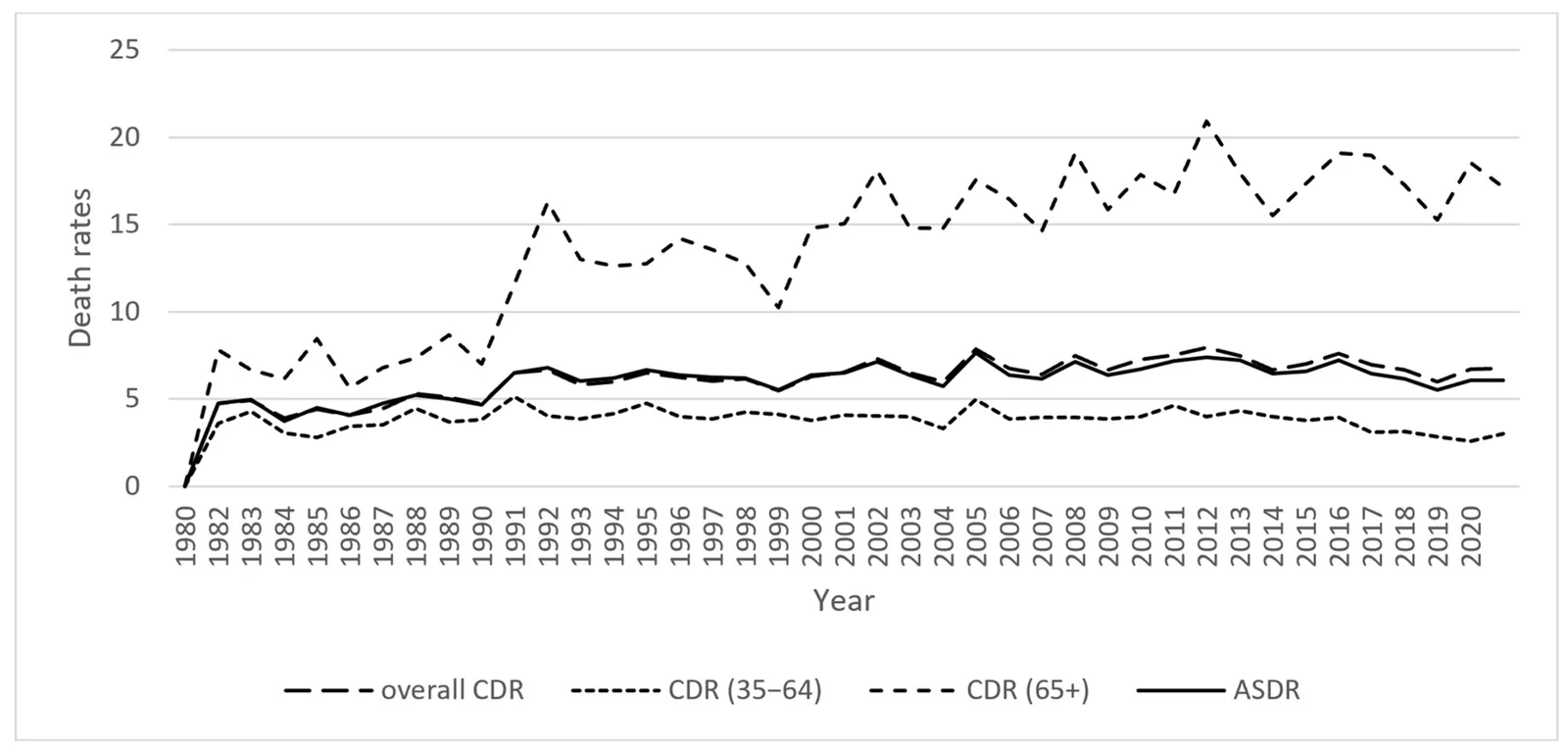
Mortality trends of crude and age-standardised death rates of melanoma in men, in Hungary 1980–2020. Abbreviations. CDR = crude death rate; ASDR = age-standardised death rate. All rates are per 100,000.

The mortality rates for melanoma were higher in men than in women (Supplementary Table S1). Visual inspection of the trends revealed that the overall CDR of melanoma in men increased until the mid-1990s (CDR = 6.51 in 1994), then stabilised until the early 2010s, and then increased again (CDR = 7.61 in 2015). The CDR in the 35–64-year-old age group remained stable throughout the study period, whereas the CDR in the ≥65 age group fluctuated but showed the most notable increase, from 7.30 to 17.13, between 1980 and 2020. ASDR increased from 3.88 to 6.08 over the same period (Figure 1).

Joinpoint analysis revealed that in the 35–64-year-old age group, the CDR significantly increased until 1990 (APC = 3.06, 95% CI = 0.74, 13.71, *p* < 0.05). In the population aged ≥65 years, an upwards trend was observed after 1987 (APC (1991–2020) = 1.26, 95% CI = 0.59, 1.83, *p* < 0.05). The increase in the ASDR was significant until 1995 (APC = 3.57, 95% CI = 2.32, 5.80, *p* < 0.05) (Table 1).

**Figure 2:**
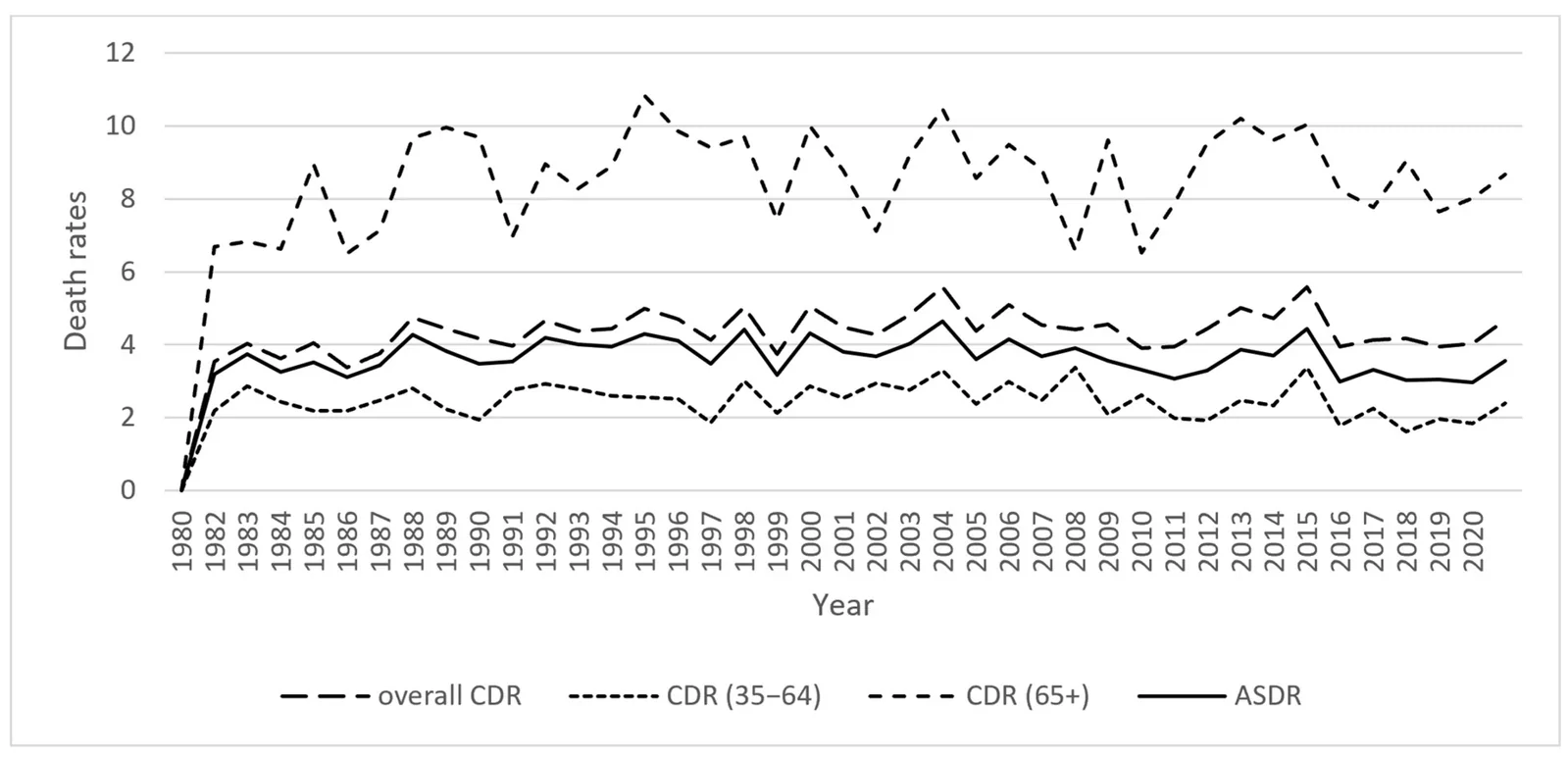
Mortality trends of crude and age-standardised death rates of melanoma in Hungary in women, 1980–2020. Abbreviations. CDR = crude death rate; ASDR = age-standardised death rate. All rates are per 100,000.

In women, the overall CDR increased slightly. The CDR in the older group increased from 6.15 to 8.66, and several years were above 10. The ASDR and CDR of the 35–64-year-old population remained stable throughout our study period (Figure 2).

Joinpoint analysis revealed a modest increase in both the ASDR (APC = 1.64, 95% CI = 0.24, 7.43, *p* < 0.05) and overall CDR trends (APC = 1.92, 95% CI = 0.58, 10.26, *p* < 0.05) until 1994. With respect to the ASDR trends, a slight downwards trend was observed thereafter (APC = −0.91, 95% CI = −2.7, 0.37, *p* < 0.05). In the 35–64-year-old age group, CDR decreased after 2005 (APC = −2.26%, 95% CI = −13.66, −0.54, *p* < 0.05), and in the older population, the CDR increased until 1988 (APC = 5.02, 95% CI = 1.09, 22.36, *p* < 0.05) (Table 1).

NMSC mortality indicators were lower in women and generally lower for NMSC than for melanoma (Supplementary Table S2). The CDR in the younger age group for women was half that for men, with an average of 1.06 for men compared with 0.50 for women, and the ASDR for men was 1.5 times that for women (ASDR = 3.42 for men vs. 1.96 for women).

**Figure 3:**
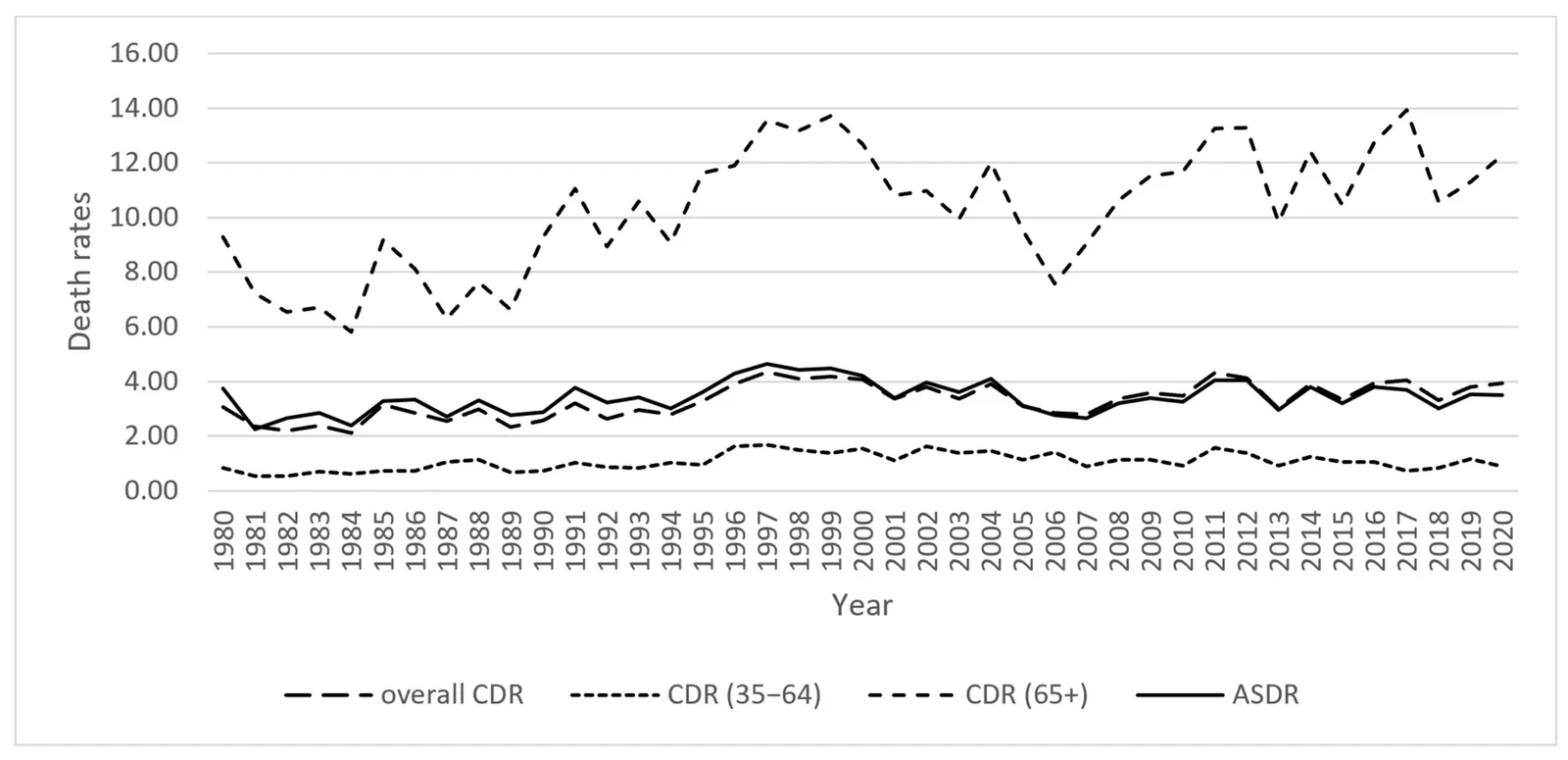
Mortality trends of crude and age-standardised death rates of NMSC in men in Hungary, 1980–2020. Abbreviations. CDR = crude death rate; ASDR = age-standardised death rate. All rates are per 100,000.

NMSC mortality trends in men showed that the overall CDR peaked between 1996 and 2000, reaching 4.33 in 1997. This aligned with the CDR of the older age group and ASDR trends, which showed the same peak period. The CDR in men under 65 years of age remained stable over this period. However, while the CDR in the ≥65 age group increased from 9.38 to 12.27 between 1980 and 2020, the ASDR, despite plateauing in 1998, decreased from 3.75 to 3.51 (Figure 3).

Joinpoint analysis revealed that the overall CDR and CDR in the ≥65 aged men, as well as the ASDR, moved in the same direction. The overall CDR significantly increased until 1998 (APC = 2.46, 95% CI = 1.22, 13.95, *p* < 0.05), and the CDR increased until 1997 (CDR above 65 years APC = 3.26, 95% CI = 1.68, 12.79, *p* < 0.05; and ASDR APC = 2.25, 95% CI = 0.69, 15.62, *p* < 0.05) (Table 2).

**Figure 4:**
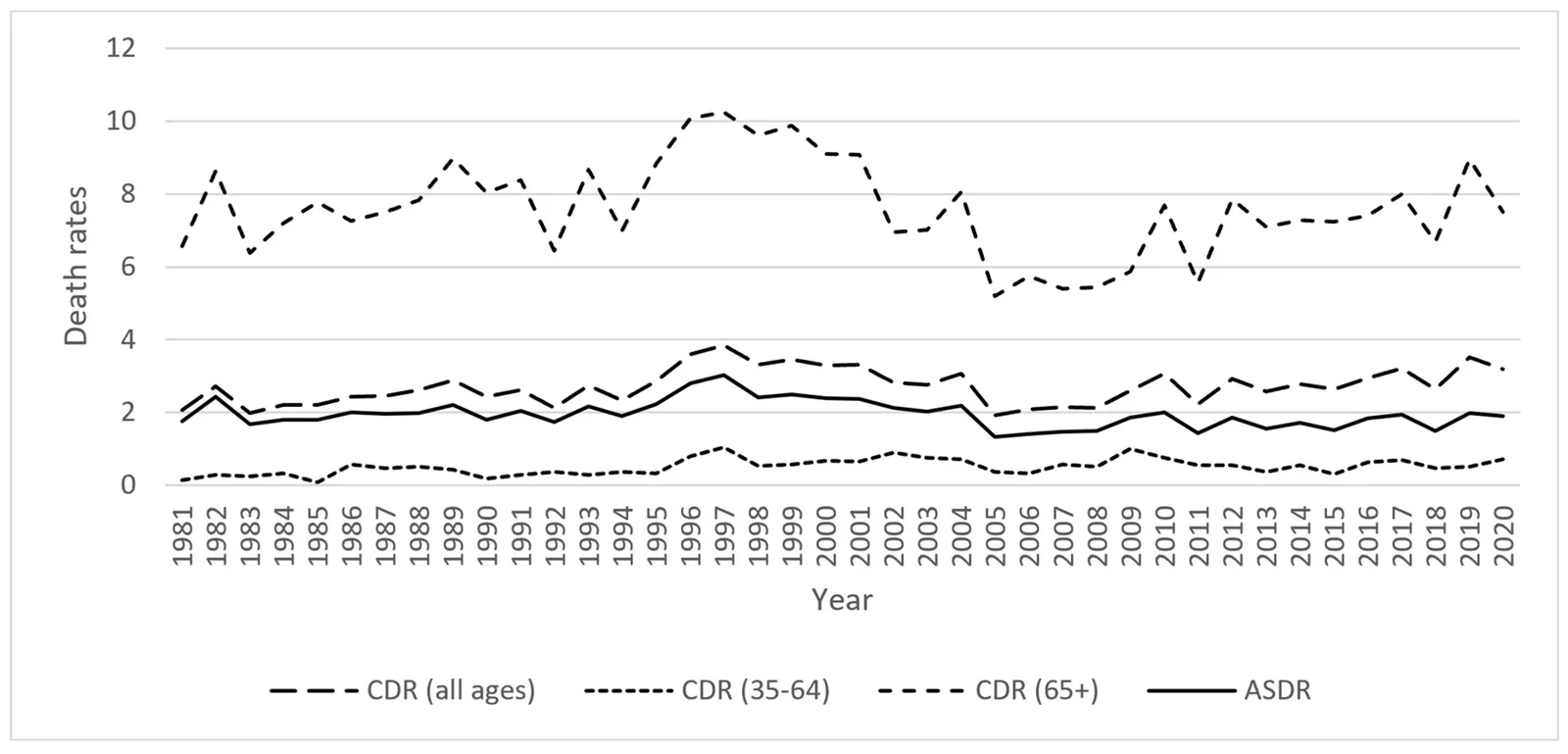
Mortality trends in crude and age-standardised death rates of NMSC in women in Hungary, 1980–2020. Abbreviations. CDR = crude death rate; ASDR = age-standardised death rate. All rates are per 100,000.

NMSC mortality trends in women revealed that the overall CDR and CDR above 65 years increased slightly, with a peak period occurring between 1996 and 2001. ASDR followed a similar pattern but generally decreased slightly over the 40-year span. The CDR in the younger group remained well below 1; the crude rate increased from 0.25 to 0.72, and occasionally rose above 1 (Figure 4).

Joinpoint trend analysis revealed a similar pattern in the overall CDR, CDR above 65 years, and ASDR: an increase until 1999 and 2001, respectively (overall CDR APC (1980–2001) = 2.38, 95% CI = 1.59, 3.49, *p* < 0.05; CDR above 65 years APC (1980–1999) = 1.87, 95% CI = 0.98, 3.05, *p* < 0.05; ASDR APC (1980–2001) = 1.65, 95% CI = 0.79, 2.98, *p* < 0.05). This was followed by a decline until the mid-2000s (overall CDR APC (2001—2005) = −10.79, 95% CI = −17.49, −3.11, *p* < 0.05, CDR above 65 years APC (1999–2006) = −7.3, 95% CI = −16.8, −3.69, *p* < 0.05, ASDR APC (2001–2005) = −11.63, 95% CI = −18.75, −3.28, *p* < 0.05). Finally, an increase was observed until 2020, which was significant only for the CDR trends (overall CDR APC (2005–2020) = 2.73, 95% CI = 1.48, 4.94, *p* < 0.05). The CDR above 65 years APC (2006–2020) = 2.74, 95% CI = 1.31, 4.84, *p* < 0.05) (Table 2).

**Figure 5:**
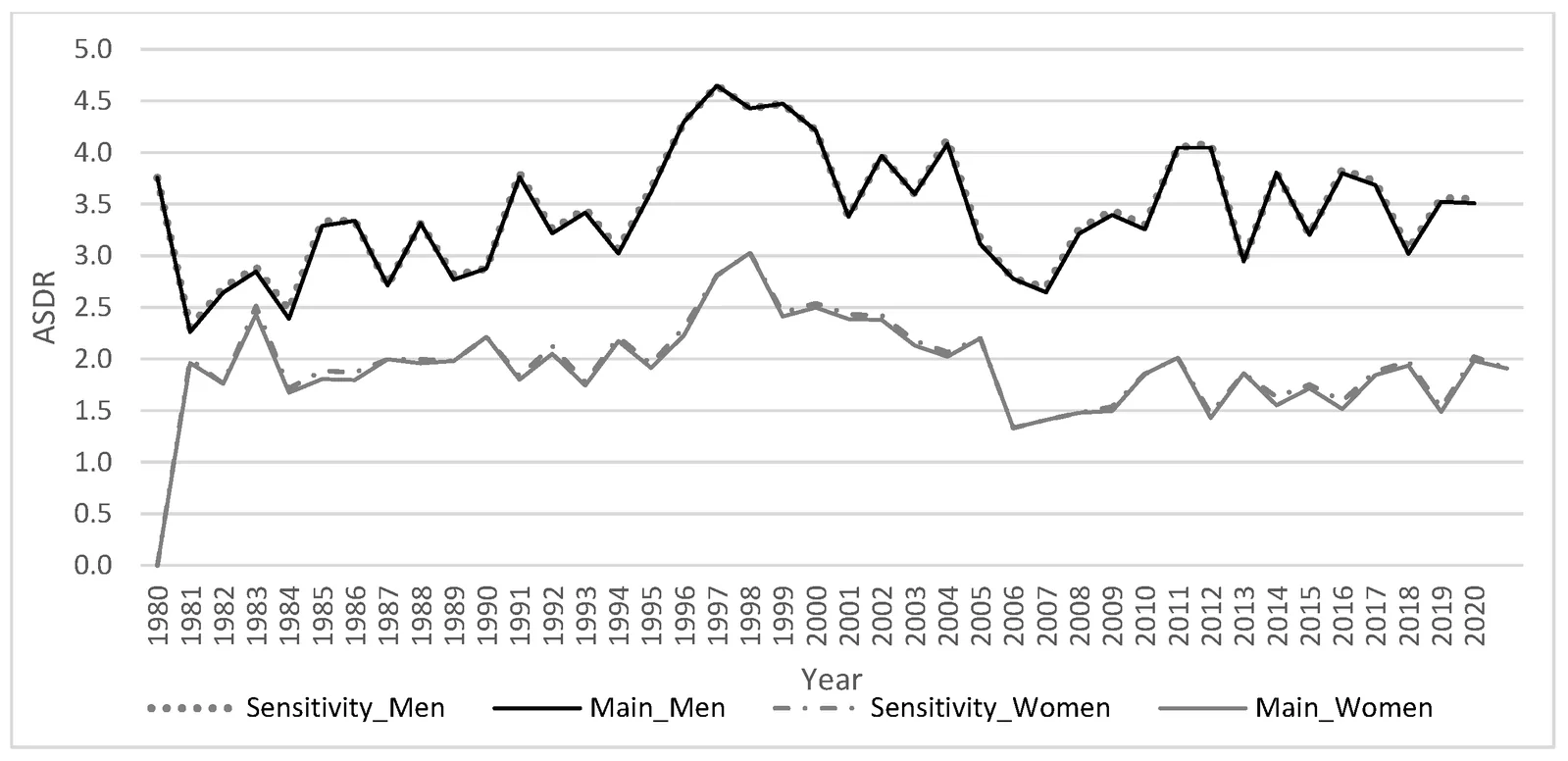
Sensitivity analysis of ASDR of melanoma and NMSC in Hungary, 1980–2020. Abbreviations. ASDR = age-standardised death rate. All rates are per 100,000. In the main analysis, substitution was performed with midpoint value of 1. In the sensitivity analysis, substitution was performed with a value of 2.

Sensitivity analysis using the alternative value of 2 in the suppressed cells showed that the differences from midpoint value substitutions were generally less than 2% with occasional deviations greater than 3%. These differences had no effect on the observed trends (Figure 5).

Compared with underlying COD coding, multiple COD records identified a greater number of deaths for both melanoma and NMSC across all age groups and in both sexes (Supplementary Table S3). The discrepancy increased markedly with age and was substantially greater for NMSC than for melanoma. Overall, multiple COD coding identified 20.3% (603/2966) more melanoma-related deaths in men and 24.6% (563/2290) more in women. For NMSC, the corresponding increases were 70.1% (1062/1516) for men and 96.9% (1336/1379) for women.

## 4. Discussion

In Hungary, melanoma mortality rates were substantially higher in men than in women. NMSC mortality remained lower overall and displayed more moderate changes.

Age-specific CDRs provided additional context for the interpretation of ASDRs. With respect to melanoma, the ASDR increased until 1994 in men and then stabilised. From 1987 to1990, there was a brief period with a sharp rise in the CDR of the older population; however, this should be interpreted with caution due to its wide CI. The crude rates in the older men steadily increased, whereas the CDR decreased in the younger age group. These findings suggest that the burden of melanoma increased between 1980 and 2020, whereas the underlying mortality risk remained largely stable, indicating that the observed increase in burden was driven primarily by population ageing rather than by an increase in age-specific mortality risk. In women, similar trends were observed. However, the older age group did not show a significant trend in the CDR. These findings may indicate partial improvements after the mid-1990s.

Similarly to melanoma, NMSC patterns suggest demographic ageing, reflected in the high CDR of individuals older than 65 years, which is consistent with earlier findings [17]. The trends observed in both the ASDR, and the CDR of the older male population moved simultaneously until 1997, which could be explained by cumulative lifetime exposure effects. There is strong evidence that cumulative lifetime exposure is associated with the incidence of NMSC but not with that of melanoma [18]. In women, mortality trends were very similar to those in men until the mid-1990s. After 2006, the Joinpoint results showed that crude rates in the older female age group increased moderately, and that the ASDR remained stable. In the younger age group, the CDR tripled; however, it remained a rare COD and it likely reflects changes in the age composition of the 35–64-year population. Melanoma had higher mortality rates than NMSC, consistent with its poorer survival; however, the healthcare burden also depends on absolute numbers. Because 36.2% of skin cancer deaths were NMSC deaths, its mortality burden should not be neglected. Our validity COD analysis indicated that NMSC was considerably more likely than melanoma to be recorded as a contributing factor rather than as the underlying cause of death.

The central and eastern European regions rank first in the melanoma mortality table. This region had a CDR of 3.3 for men and 3.0 for women, and an ASDR using the World Standard Population (WSP 1960) of 2.1 for men and 1.4 for women in 2018 [19]. Our results showed that the overall CDR and ASDR exceeded the regional rates in 2020, although the results are not directly comparable because the analysis included different age groups and used different standard populations. In the Globocan database, the ASDRs (using WSP 1976) for the ages 35–85+ show that Hungary had an average rate within the region in 2022 [20]. These findings are in line with the finding of Koczkodaj et al. (2023) [12], who calculated the ASDR rates for individuals aged 45–75 years (5.6 in men and 3.0 in women). Koczkodaj et al. (2023) reported a decrease in the ASDR of melanoma among those aged 45–75 in Hungary from 1970 to 2020 [12]. Our earlier results using the HCSO’s cause-specific mortality database indicated stabilisation in the ASDR (standardised by ESP 2013) in the 45–64-year-old and the older generations for the period 2001–2020, and here we reported that most of the increase occurred during the decade after the transition [21]. It must be acknowledged that using death certificates gave us a slight deviation in the number of individuals who died of melanoma between 2001 and 2020 (5788 vs. 5917).

The literature concerning both the mortality and incidence of NMSC is scarce. Dai and colleagues (2026), who studied temporal trends from 1990 to 2021, reported that the overall ASDR for NMSC decreased from 1.59 to 0.67, corresponding to an annual APC (AAPC) of −3.08 (95% CI = −3.32–2.83). Although methodologically not comparable, they estimated the ASDR to be 1.45 in 1990 and 0.75 in Hungary (AAPC = −2.02 (−2.31–1.73)) [22].

The trends observed in the older age group could be explained by the marked demographic transition Hungary underwent between 1980 and 2020, which could have affected the observed CDR trends. First, the population decreased by 9% over the study period. Second, classical population ageing characterised this period. In 1980, 13.5% of the population was older than 65 years; this percentage increased to 15.1% in 2001 and reached 19.8% in 2020 [23]. This translated into an extra half a million high-risk persons in 2020. Moreover, life expectancy has improved. In men, it increased from 65.5 years to 72.2 years, and in women, it increased from 72.7 years to 78.7 years [24].

Several hypotheses may explain the observed findings; however, the present dataset does not provide evidence to directly evaluate or confirm the underlying mechanisms. Therefore, the explanations in the next three paragraphs remain speculative.

Historically, Hungary faced worsening mortality rates during the transition period until the mid-1990s, reflected in a decline in life expectancy at birth [25]. Overall cancer mortality rates increased sharply after the 1990s and peaked in 1995. This phenomenon was referred to as state socialist mortality syndrome, which particularly affected working-age men, aged 40 to 54 at that time, and could explain the disproportionate cohort effect in men [26].

In Hungary, oncological care has undergone significant improvements since 1990. Manuals on oncology diagnostics and care were issued, the Institute of Oncology was established in 1993, and the National Cancer Control Program was funded in 2000 [27]. After the transition, tighter workforce regulation and enforcement came into effect, particularly in the agricultural sector. Protective clothing is designed to minimise dermal exposure to pesticides, with reduced solar exposure being an additional advantage. Despite improvements in infrastructure, care, and diagnostics, Hungary remained affected by persistent high cancer mortality and even exceeded the EU-27 average by 32% in 2021. The contributing factors are often high alcohol and tobacco consumption, overweight status, late diagnosis, and health inequality, especially those related to education level [28,29]. Since 1990, dermatology has undergone substantial privatisation [30]. This disproportionately benefits those who can afford it, while shutting out the most vulnerable.

In relation to changes in exposure to ultraviolet radiation, sunbeds were introduced in Hungary in the late 1980s. A significant historical change that likely affected UV exposure trends was the opening of borders, which facilitated international travel. The emergence of a new middle class was indeed able to afford foreign travel [31]. Both factors are crucial risk elements because they involve short, intense bursts of ultraviolet radiation, known as intermittent exposure, which has been associated with melanoma development [32,33].

Our study has several limitations. The analysed time series ends in 2020, which limits the contemporary relevance of the findings. As the manuscript was prepared several years later, changes in melanoma treatment patterns, demographic characteristics, healthcare access, and clinical practice occurring after 2020 are not captured. Consequently, the results may not fully reflect the current epidemiology and management of melanoma. Our approach to suppressing small cell counts (1–2 deaths) in selected age groups (35–44 and 45–54) for the ASDR calculation introduced uncertainty into age-specific mortality estimates. A sensitivity analysis revealed a minimal effect on the overall trends, but the true value remains unknown. Our decision to use the 1976 standard population to account for differences in the age structure of the population might not accurately reflect the age structure of the Hungarian population over the entire span of the study. The age categories in the CDR are broad and may mask age-specific patterns. When a wide CI was observed, interpretation must be approached with caution. Wide CIs are likely to be due to high year-to-year variability and small underlying case numbers. Furthermore, the Joinpoint analysis assumes a linear trendline, but in reality, the trend is often more gradual [16]. It is also uncertain whether the coroner captured all the cases. Assuming that the COD was ascertained based on previous diagnosis or postmortem examination, diagnostic error remains negligible. This is due to diagnostic uncertainty rather than diagnostic skills, according to Elder et al. (2023) [34]. Wéber et al. assessed the data quality of incident cancer cases at the Hungarian National Cancer Institute and found that 54.8% of melanoma cases and 80% of NMSC cases were morphologically verified, but only 0.1% of reported incident cases were derived from death certificates only [35]. Finally, BCC and SCC were not separately recorded, and the death rates of these subcategories could not be estimated. Recent (yet unpublished) research indicated that BCC accounted for 75% of all NMSC incident NMSC cases between 2000 and 2023 in Hungary [36]. Global estimates show that SCC is responsible for the majority of NMSC deaths and its burden has been underestimated [37,38].

A major strength of this research is its extensive study period, which enabled the collection of a robust sample size. It also addresses NMSC mortality, which is often overlooked in morbidity and mortality statistics. ASDR, together with age-specific CDR, enhanced the interpretability of the overall trends. Lastly, it explicitly addresses the methodological challenge posed by suppressed small cell counts (1–2 deaths) by applying a transparent substitution approach, combined with sensitivity analyses.

## 5. Conclusions

We conclude that melanoma mortality trends showed partial stabilisation or improvement after the mid-1990s, particularly in terms of age-standardised rates. However, mortality remained high and, in some cases, increased among older adults, especially among men. The NMSC mortality burden was largely concentrated in the population aged 65 years or older, as shown by consistently higher crude mortality rates in this age group, and a renewed increase among older women after the mid-2000s.

While melanoma has attracted much attention globally, including in Hungary, the incidence of NMSC is often underreported both globally and in Hungary. Despite being the most common cancer, its mortality is rarely studied [6]. We found that NMSC also placed a burden on healthcare between 1980 and 2020. Overall, our research provides information on overall skin cancer mortality rates and confirms that NMSC should be included in statistics, especially for calculating disease burden.

The findings also point to the need for stronger prevention and early detection efforts, particularly among adults aged 65 and older. Because skin cancer risk accumulates over the life course, prevention should focus on increasing awareness of the harmful effects of ultraviolet radiation, encouraging simple sun-protective habits (e.g., sunscreen use, reducing midday sun exposure, and adequate clothing), and helping individuals recognise suspicious skin changes. This could improve outcomes. We attempted to provide explanations for the observed trends; however, further research is warranted.

## Figures and Tables

**Table 1:** Joinpoint analysis of melanoma mortality trends in Hungary, 1980–2020.

Men					Women				
Segment	Years	APC (%)	95% CI		Segment	Years	APC (%)	95% CI	
*Crude rates—overall*									
1	1980–1991	3.52	−1.51	14.29	1	1980–1994	**1.92 ^#^**	0.58	10.26
2	1991–2011	1.22	−7.51	9.07	2	1994–2020	−0.30	−3.53	0.22
3	2011–2020	−1.50	−11.36	5.08					
*35–64 years*									
1	1980–1990	**3.06 ^#^**	0.74	13.71	1	1980–2005	0.55	−0.31	8.45
2	1990–2013	−0.15	−2.10	0.79	2	2005–2020	**−2.26 ^#^**	−13.66	−0.54
3	2013–2020	**−5.72 ^#^**	−15.51	−1.72					
*65 years old or above*									
1	1980–1987	−1.41	−12.20	2.77	1	1980–1988	**5.02 ^#^**	1.09	22.36
2	1987–1991	**18.81 ^#^**	8.18	31.00	2	1988–2020	−0.22	−1.78	0.28
3	1991–2020	**1.26**	0.59	1.83					
*ASDR*									
1	1980–1994	**3.57**	2.32	5.80	1	1980–1994	**1.64**	0.25	7.43
2	1994–2020	0.00	−0.69	0.50	2	1994–2020	**−0.91**	−2.70	−0.37

Abbreviations. APC = Annual Percent Change; ASDR = age-standardised death rate. All rates are per 100,000. **bold** represents *p* < 0.05. ^#^ represents wide CI.

**Table 2:** Joinpoint analysis of NMSC mortality trends in Hungary, 1980–2020.

Men					Women				
Segment	Years	APC (%)	95% CI		Segment	Years	APC (%)	95% CI	
*Crude rates—overall*									
1	1980–1998	**2.46 ^#^**	1.23	13.95	1	1980–2001	**2.38**	1.59	3.49
2	1998–2020	0.09	−8.19	0.98	2	2001–2005	**−10.79**	−17.49	−3.11
					3	2005–2020	**2.73**	1.48	4.94
*35–64 years*									
1	1980–1999	**4.88**	3.07	7.82	1				
2	1999–2020	**−2.06**	−4.28	−0.58	2				
*65 years or older*									
1	1980–1997	**3.25 ^#^**	1.68	12.79	1	1980–1999	**1.87**	0.98	3.05
2	1997–2020	0.09	−5.01	1.06	2	1999–2006	**−7.30**	−16.80	−3.69
					3	2006–2020	**2.74**	1.31	4.84
*ASDR*									
1	1980–1997	**2.25 ^#^**	0.69	15.62	1	1980–2001	**1.65**	0.79	2.98
2	1997–2020	−0.68	−9.36	0.27	2	2001–2005	**−11.63**	−18.75	−3.28
					3	2005–2020	1.24	−0.15	4.58

Abbreviations. APC = Annual Percent Change; ASDR = age-standardised death rate. All rates are per 100,000. **bold** represents *p* < 0.05. ^#^ represents wide CI.

## Data Availability

All data included in this paper were obtained from the Hungarian Central Statistical Office (HCSO). To protect individual privacy, raw data was not disclosed to the researchers. Instead, aggregated data was made available in the HCSO’s research room. Only the analysis results were released after strict adherence to data protection protocols.

## Supplementary Materials

The following supporting information can be downloaded at https://www.mdpi.com/article/10.3390/cancers18162533/s1, Table S1: Crude and age-standardised death rates of melanoma (ICD10 C43) in Hungary, 1980–2020; Table S2: Crude and age-standardised death rates of NMSC (ICD10 C44) in Hungary, 1980–2020; Table S3: Skin cancer deaths identified from primary and multiple cause-of-death records by sex.

## Abbreviations

APC	Annual Percent Change
ASDRs	Age-standardised death rates
BCC	Basal cell carcinoma
CDRs	Crude death rates
CI	Confidence interval
COD	Cause of death
DALY	Disability-adjusted life years
ESP	European Standard Population
GDPR	General Data Protection Regulation
HCSO	Hungarian Central Statistical Office
NMSC	Non-melanoma skin cancer
NUTS	Nomenclature of Territorial Units for Statistics
SCC	Squamous cell carcinoma
WSP	World standard population

## References

[1] Rudolph C., Schnoor M., Eisemann N., Katalinic A. (2015). Incidence Trends of Nonmelanoma Skin Cancer in Germany from 1998 to 2010. JDDG J. Dtsch. Dermatol. Ges..

[2] Anselmo Lima C., Sampaio Lima M., Maria Da Silva A., Prado Nunes M.A., Macedo Lima M.M., Oliveira Santos M., Lyra D., Kleber Alves C. (2018). Do Cancer Registries Play a Role in Determining the Incidence of Non-Melanoma Skin Cancers?. Eur. J. Dermatol..

[3] Urban K., Mehrmal S., Uppal P., Giesey R.L., Delost G.R. (2021). The Global Burden of Skin Cancer: A Longitudinal Analysis from the Global Burden of Disease Study, 1990–2017. JAAD Int..

[4] Leiter U., Keim U., Eigentler T., Katalinic A., Holleczek B., Martus P., Garbe C. (2017). Incidence, Mortality, and Trends of Nonmelanoma Skin Cancer in Germany. J. Investig. Dermatol..

[5] Perera E., Sinclair R. (2013). An Estimation of the Prevalence of Nonmelanoma Skin Cancer in the U.S. F1000Research.

[6] European Academy of Dermatology (2023). Non-Melanoma Skin Cancer Killing More People than Melanoma. New Study Finds.

[7] Bray F., Laversanne M., Sung H., Ferlay J., Siegel R.L., Soerjomataram I., Jemal A. (2024). Global Cancer Statistics 2022: GLOBOCAN Estimates of Incidence and Mortality Worldwide for 36 Cancers in 185 Countries. CA Cancer J. Clin..

[8] OECD (2021). State of the Health in the EU, Hungary—Country Health Profile.

[9] Parrag P., Wéber A., Liszkay G., Nagy P., Kásler M., Polgár C., Kenessey I. (2022). A Melanóma Hazai Morbiditási És Mortalitási Helyzete a XXI. Század Első Két Évtizedében [Hungarian Situation of Melanoma Incidence and Mortality in the First Two Decades of 21st Century]. Magy. Onkol..

[10] Várnai M., Kiss Z., Gyulai R., Oláh J., Holló P., Emri G., Csejtei A., Kenessey I., Benedek A., Polányi Z. (2021). Improving Quality Indicator of Melanoma Management—Change of Melanoma Mortality-to-Incidence Rate Ratio Based on a Hungarian Nationwide Retrospective Study. Front. Oncol..

[11] Gubán R., Parrag P., Kispál M.T., Czirbesz K., Danyi T., Kenessey I., Liszkay G. (2025). Characterization of Melanoma in Hungary Based on a Retrospective Single-Center Study Between 2001 and 2018. Cancers.

[12] Koczkodaj P., Sulkowska U., Didkowska J., Rutkowski P., Mańczuk M. (2023). Melanoma Mortality Trends in 28 European Countries: A Retrospective Analysis for the Years 1960–2020. Cancers.

[13] Stier A., Páldy A. (2021). Temporal Changes in Skin Cancer Epidemiology in Hungary (2001–2015). Ann. Dip. Metod. Model. L’Econ. Territ. Finanz..

[14] Központi Statisztikai Hivatal [Hungarian Central Statistical Office] (2024). Területi Atlasz—Járások [Regional Atlas—Districts].

[15] Pace M., Lanzieri G., Glickman M., Grande E., Zupanic T., Wojtyniak B., Gissler M., Cayotte E., Agafitei L. (2013). Revision of the European Standard Population.

[16] National Cancer Institute Division of Cancer Control and Population Sciences Joinpoint Trend Analysis Software. https://surveillance.cancer.gov/joinpoint/.

[17] Papp B.T., Toplenszky K., Ócsai H., Csányi I., Kemény L., Gyulai R., Oláh J., Baltas E. (2025). Ten Years of Euromelanoma in Hungary: Nationwide Trends and Risk Factors for Skin Cancer in Central–Eastern Europe. Cancers.

[18] International Agency for Research on Cancer (IARC) (2012). IARC Monographs on the Evaluation of Carcinogenic Risks to Humans: Volume 100D, Radiation.

[19] Arnold M., Singh D., Laversanne M., Vignat J., Vaccarella S., Meheus F., Cust A.E., de Vries E., Whiteman D.C., Bray F. (2022). Global Burden of Cutaneous Melanoma in 2020 and Projections to 2040. JAMA Dermatol..

[20] International Agency for Research on Cancer Cancer Today: Data Visualization (GLOBOCAN 2022) [Online Database]. Global Cancer Observatory. https://gco.iarc.who.int/today/en/dataviz/pie?mode=population&group_populations=0.

[21] Stier Á., Kenessey I., Páldy A. (2026). Long-Term Changes in Melanoma Epidemiology in Hungary: Who Are the High-Risk Groups, and Where Should We Concentrate Prevention Programs?. BMC Public Health.

[22] Dai Y., Liu X., Tang H. (2026). Global Regional and National Burden of Nonmelanoma Skin Cancer from 1990 to 2021 Based on the Global Burden of Disease 2021 Study. Discov. Oncol..

[23] Hungarian Central Statistical Office Népesség Korév És Nem Szerint, Január 1. (1980–)* [Population According to Age and Sex, from 1. January 1980] (STADAT Table I_wdsd009). https://www.ksh.hu/docs/hun/xstadat/xstadat_eves/i_wdsd009.html.

[24] Hungarian Central Statistical Office Népesség, Népmozgalom [Population, Vital Statistics] (Table H_wdsd001b). https://www.ksh.hu/docs/hun/xstadat/xstadat_hosszu/h_wdsd001b.html.

[25] Bálint L., Kovács K., Monostori J., Őri P., Spéder Z. (2015). Mortality. Demographic Portrait of Hungary 2015.

[26] Carlson E., Hoffmann R. (2011). The State Socialist Mortality Syndrome. Popul. Res. Policy Rev..

[27] Kásler M., Ottó S. (2008). European and national tasks of oncology [Európai És Hazai Kihívások Az Onkológiában]. Magy. Onkol..

[28] Organisation for Economic Co-Operation and Development (OECD), European Commission (2025). Hungary Country Cancer Profile 2025. European Cancer Inequalities Registry.

[29] Ádány R., Juhász A., Nagy C., Burkali B., Pikó P., McKee M., Oroszi B. (2024). Discrepancies between the Spatial Distribution of Cancer Incidence and Mortality as an Indicator of Unmet Needs in Cancer Prevention and/or Treatment in Hungary. Cancers.

[30] Kornai J., Eggleston K. (2001). Choice and Solidarity: The Health Sector in Eastern Europe and Proposals for Reform. Int. J. Health Care Finance Econ..

[31] Szelényi I. (1998). Theories of the New Class.

[32] Koumaki D., Evangelou G., Gregoriou S., Kouloumvakou S., Manios A., Katoulis A., Zacharopoulos G., Chernyshov P., Papadakis M., Kassotakis D. (2024). Skin Cancer Knowledge, Sun Exposure, Photoprotection Behavior, and Perceived Barriers Associated with Skin Cancer Types in a Greek Cohort: A Cross-Sectional Study on the Island of Crete. Cancers.

[33] Wunderlich K., Suppa M., Gandini S., Lipski J., White J.M., Del Marmol V. (2024). Risk Factors and Innovations in Risk Assessment for Melanoma, Basal Cell Carcinoma, and Squamous Cell Carcinoma. Cancers.

[34] Elder D.E., Eguchi M.M., Barnhill R.L., Kerr K.F., Knezevich S.R., Piepkorn M.W., Reisch L.M., Elmore J.G. (2023). Diagnostic Error, Uncertainty, and Overdiagnosis in Melanoma. Pathology.

[35] Wéber A., Mery L., Nagy P., Polgár C., Bray F., Kenessey I. (2022). Evaluation of Data Quality at the Hungarian National Cancer Registry, 2000–2019. Cancer Epidemiol..

[36] Wéber A., Szatmári I., Dobozi M., Nagy P., Dank M., Kenessey I. Hungarian Trends in the Incidence of Non-Melanoma Skin Cancer and the Impact of COVID Pandemic. Proceedings of the IARC 60th Anniversary.

[37] Apalla Z., Nashan D., Weller R.B., Castellsagué X. (2017). Skin Cancer: Epidemiology, Disease Burden, Pathophysiology, Diagnosis, and Therapeutic Approaches. Dermatol. Ther..

[38] Wehner M.R. (2020). Underestimation of Cutaneous Squamous Cell Carcinoma Incidence, Even in Cancer Registries. JAMA Dermatol..

